# Upper Limb Kinematics of Handwriting among Children with and without Developmental Coordination Disorder

**DOI:** 10.3390/s22239224

**Published:** 2022-11-27

**Authors:** Amani Abu-Ata, Dido Green, Ran Sopher, Sigal Portnoy, Navah Z. Ratzon

**Affiliations:** 1 Department of Occupational Therapy, School of Health Professions, Tel Aviv University, Tel Aviv 6997801, Israel; 2 Department of Rehabilitation, School of Health and Welfare, Jönköping University, 553 18 Jönköping, Sweden; 3 Department of Biomedical Engineering, Faculty of Engineering, Tel Aviv University, Tel Aviv 6997801, Israel

**Keywords:** DCD, handwriting, legibility, 3D motion analysis, kinematics

## Abstract

Background: Children with developmental coordination disorder (DCD) often experience difficulties with handwriting legibility and speed. This study investigates the relationship between handwriting and upper limb kinematics to characterize movement patterns of children with DCD and typically developing (TD) children. Methods: 30 children with and without DCD matched for age, gender, and parent education were compared across handwriting abilities using a standardized handwriting assessment of both copied and dictated tasks (A-A Handwriting). The 3D motion capture system (Qualysis) was used to analyze upper limb kinematics and characterize movement patterns during handwriting and contrasted with written output. Results: Children with DCD wrote fewer legible letters in both copying and dictation. Children with DCD also showed poor automatization of key writing concepts. Atypical wrist postures were associated with reduced legibility for children with DCD (F (1,27) 4.71, *p =* 0.04, p-η^2^ = 0.15); whereas for TD children, better legibility was associated with greater variations in movement speed, particularly of the wrist (*rho =* −0.578, *p* < 0.05). Conclusion: Results reflect different movement parameters influencing handwriting in children with DCD. An improved understanding of the movement characteristics during handwriting of these children may assist intervention design.

## 1. Introduction

The ability to write by hand is one of the most fundamental yet complex skills acquired during childhood and forms one of the main occupations of children at school [1]. A number of components contribute to the ability to write. These not only include cognitive and perceptual processes and an understanding of the representational nature of graphic forms, but importantly require different aspects of movement control, both kinesthetic and ergonomic [2].

Developmental coordination disorder (DCD), a common neurodevelopmental disorder affecting the acquisition of functional motor skills, frequently impacts handwriting ability [3]. Problems with the acquisition of handwriting form one of the most common reasons for referral to pediatric occupational therapy [4,5]. Searches for the mechanisms underpinning deficits in handwriting proficiency in children with DCD have indicated poor visual motor integration and memory, over-reliance on feedback control, reduced ability to use control strategies for end-point accuracy, and/or deficits in motor imagery or action representation [6,7,8,9]. However, these visual perceptual/visual-motor components have not been directly linked to the problems in handwriting (legibility and or speed) experienced by children with DCD [10]. More recently, using a hand dynamometer and digitizing tablet, Prunty et al. [11] showed that neither biomechanical issues of grip strength and pen pressure were associated with the handwriting difficulties of these children.

In typically developing (TD) children, hand fluency and automaticity have been shown to be associated with writing composition; freeing up cognitive resources for ideas and topics, as evidenced through comparisons of writing productivity in self-initiated versus topic-directed tasks [12]. Thus the poor automatization of movements has been hypothesized as a primary deficit for children with DCD influencing motor control, speed, and fluency in writing complex characters, particularly when there are additional cognitive demands for writing compositions [13,14].

The control of movement and movement variability, described by Bernstein [15] as the control of the ‘degrees of freedom’ problem, is achieved through the use of coordinated synergies [16]. Reduced muscle synergies may also contribute to poor pencil control for children with DCD (See [17] for discussion of muscle synergies in motor behaviors). Utilizing a developmental contingency model, Green et al. [18] conceptualized that variability in the performance of children with DCD, results from a dynamic interaction between different individual behavioral (including emotional states), cognitive capacities, demands of the task, and the environmental context. Thus, motion parameters and subsequent performance may be influenced as much by differing task demands as individual capacity [19].

When observing the task of handwriting, the evidence points towards a distinction between different types of handwriting styles (e.g., printed versus cursive) and tasks (e.g., copying versus dictation) and their influence on handwriting performance [10,20,21,22,23]. The differential movement parameters (joint positions, accelerations/decelerations, and overall speed) that may influence performance in these different tasks have yet to be explored. Research to date has neither considered the different kinematic parameters involved in performing the typical handwriting tasks of copying or dictation, nor how these may differ between children with and without DCD. Further, with a clinical focus to encourage children with handwriting deficits to acquire keyboard skills and concern over the longer-term consequences of atypical postures and potential musculoskeletal disorders [24], there is a need to define the movement kinematics of handwriting under different conditions (e.g., copying and dictation) in order to contrast the ergonomics of handwriting and keyboarding.

This study aims to quantify differences in handwriting between children with and without DCD. More specifically, the aim of this study is to investigate the performance and movement characteristics, including variability, during handwriting of children with DCD compared with TD children. We thus aim to quantify the differences in performance between children with DCD and TD children on writing components of speed and legibility across two different writing tasks of (i) copying and (ii) dictation (differing in demands of visual-motor control) and identify corresponding movement characteristics. We hypothesized that children with DCD would show less productivity (slower/less written) and poorer legibility associated with atypical postures and either greater variability of movement range across joints or restrictions of distal movement. We explored differences between visual- and auditory- motor integration without an opinion as to the relative advantages for copying (visual) or dictation (auditory) stimuli. For this purpose, we used 3D motion tracking, in an exploratory study, to define joint kinematics of children with DCD and TD children and correlate these with performance in writing tasks of copying and dictation. The expansion of research in this field will raise understanding of the parameters of motor control associated with skilled handwriting in children with and without movement difficulties.

## 2. Materials and Methods

The study was approved by the university ethics committee with informed consent obtained from parents and children prior to participation in the study. Participants were free to withdraw from the study at any time for any reason.

### 2.1. Participants

The study population consisted of children aged 7 to 9 years, studying in classes in regular education. Children diagnosed with DCD (*n* = 15) by the attending pediatrician according to DSM-IV criteria from a regional child development centre were sent invitations to participate in the study. Age-matched typically-developed peers were recruited through an associated community advertisement for volunteers. Exclusion criteria for both groups were left-hand dominancy, any physical, neurological or metabolic disturbance (except for DCD), and scoring below the 16th percentile on the Conners’ Parent Rating Scale (CPRS; [25]). Information on estimated writing hours per day and parental education levels were also obtained.

### 2.2. Measures

The CPRS was used to identify attention deficits that may impact skills or performance and rule out the possibility of attention deficit hyperactive disorder (ADHD). The CPRS shows good predictive validity to distinguish populations with ADHD from typical populations [25]. The short version used in this study consisted of 48 questions measured on an ordinal scale 0–3 (0-not observed, 3-observed a lot) with higher scores indicating more ADHD symptoms [26]. In the current study scores above 40 (below 16th percentile) were considered indicative of hyperactive symptoms and these children were excluded.

Developmental Coordination Disorder Questionnaire-07 (DCDQ; [27]), a 15-item parent report tool, was used to screen for any movement difficulties in children, and exclude children who may be at risk of DCD in the control group and confirm the presence of functional movement difficulties in children with DCD. Total scores range between 15 and 75, with higher scores indicating better motor function. Scores below 48 are indicative of movement difficulties. The questionnaire has good internal reliability and external validity and shown good sensitivity (85%) and specificity (71%) as a screening tool for coordination difficulties in children [27].

Movement Assessment Battery for Children-2nd Edition (MABC-2; [28]) was used to determine the extent of movement difficulties in the group of children previously diagnosed with DCD. The MABC-2 is considered a gold standard for determining Criterion A (presence of significant movement difficulties), for the diagnosis of DCD and contains 8 subtests across 3 domains: manual dexterity, aiming and catching, and balance with standard scores, and percentiles obtained across ages. The MABC-2 has good test-retest and inter-rater reliability and concurrent validity [29]. Scores below the 5th percentile along with a score <48 on the DCDQ were used to confirm diagnosis of DCD.

The Hebrew Handwriting Evaluation (HHE) [30], designed for children aged 6–10 years, was used to assess different aspects of Hebrew handwriting. The paragraph used in the copying and dictating task contains all the letters in the Hebrew alphabet, and includes 30 words and 107 letters in total. Two types of handwriting outcome variables are extracted. The first are legibility scores, reflecting the ability to read what is written and organization of text and rated on a scale of 1–4, with higher scores reflecting poorer ability (more problems in legibility). These consist of global legibility, and more specific elements involving unrecognizable letters, spatial arrangement and alignment of the written text. Measurements also involve calculating spaces between words, sizes of letters, letter formation, and maintenance of the margins using a set calibrated instrument. Scoring descriptors are provided; for example ‘not proper letter formation’ would be indicated if different parts of the letter were not connected when they need to be. The second type of variable measures handwriting efficiency and includes handwriting speed. Scoring of the HHE is divided as follows: (a) handwriting speed is measured by the number of letters produced per minute and the time it takes to write the whole text; (b) legibility scores are measured on a 4-point Likert scale, from the most legible (1) to the least legible (4), which refers to the overall clarity of the handwriting product and ability of the reader to identify the letters. A total HHE legibility score is obtained by summing legibility and spatial arrangement components with scores ranging from 7 to 24 (higher scores representing poorer performance). In addition, other measurements relating to writing productivity (text produced in a specific time), such as the number of letters erased, are recorded. High internal consistency (α = 0.81) and inter-rater reliability (ranging from r = 0.75 to r = 0.79) are reported [30]. Deletions (erasing) are counted separately. Construct validity of the HHE has been established by demonstrating significant differences between the performance of children with proficient and poor handwriting across school grades (second and third). No significant differences have been found for gender [30]. Ten percent of HHE samples were rated by an independent therapist, blinded to group allocation.

**Figure 1:**
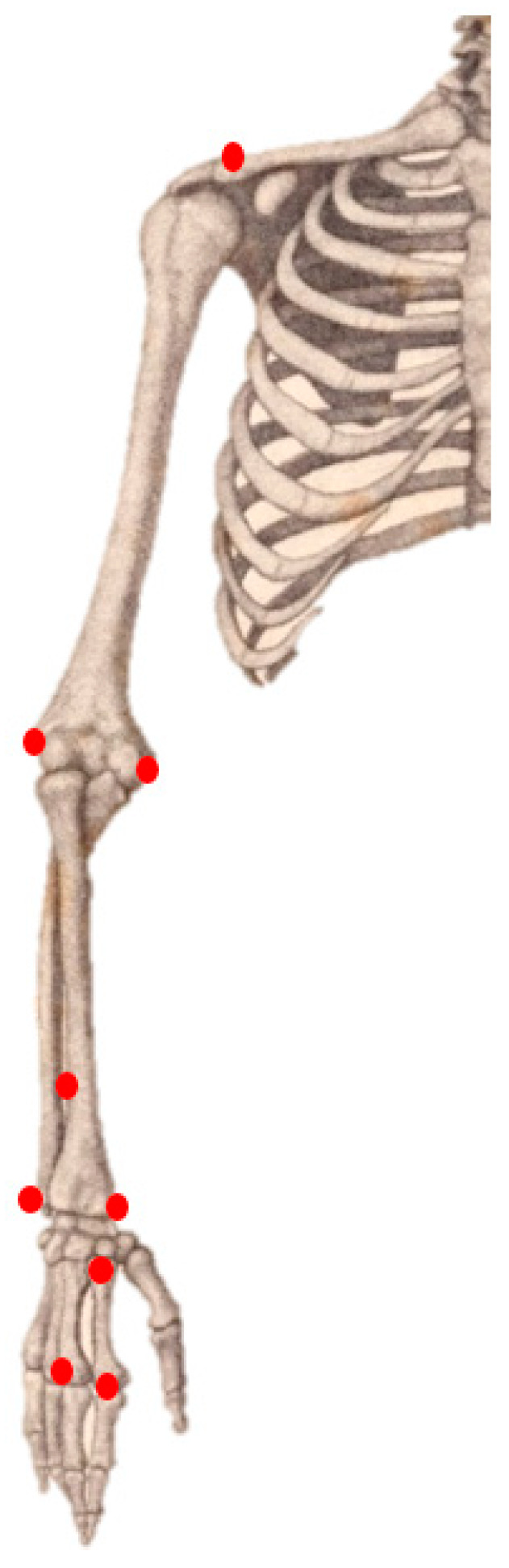
Placement location of the nine markers: the right upper arm (one marker on the acromion and two markers on the medial and lateral aspects of the distal humerus), forearm (two markers on the ulnar and radial styloid processes, and a third tracking marker on the distal third dorsal aspect of the forearm), and hand (proximal and distal heads of the 2nd metacarpal and distal head of the 3rd metacarpal) of each subject.

Kinematic Measurements—The Qualisys 3D motion capture system (Qualisys, Göteborg, Sweden) was used to analyze upper limb kinematics and characterize movement patterns in copying and dictated tasks. Six Oqus 300 cameras, positioned around the workstation, tracked the 3D coordinates of the markers at a sampling rate of 100 Hz. The motion capture system was calibrated before each trial for resolutions higher than 1 mm. Nine passive-reflective spherical (7 mm) markers (Figure 1) were placed on specific anatomical landmarks of the right upper arm (one marker on the acromion and two markers on the medial and lateral aspects of the distal humerus), forearm (two markers on the ulnar and radial styloid processes, and a third tracking marker on the distal third dorsal aspect of the forearm), and hand (proximal and distal heads of the 2nd metacarpal and distal head of the 3rd metacarpal) of each subject. These markers were used to optimize accurate capture of small movements consistent with other studies tracking activities of daily living such as drawing [31]. Elbow flexion–extension was calculated as the angle between the forearm in relation to the upper-arm; wrist flexion–extension and radial ulnar deviation were calculated as the angle of the hand in relation to the forearm.

A reference model for anatomic scaling of each subject was acquired, using a 10 s static posture recording. Missing data during the dynamic trials were gap-filled using the Qualisys Track Manager (QTM) algorithm, in which missing marker information is reconstructed from the intercorrelations between marker coordinates, only if <20% missing and visual tracking of motion traces were logically fitted. The 3D coordinate data were exported into a custom MATLAB^®^ script, where they were low-pass filtered using a 7th order Butterworth filter with a cut-off frequency of 5 Hz. The minimum, maximum, and ranges of the elbow, wrist angles, and angular accelerations were computed (with wrist flexion and ulnar deviation, as well as elbow flexion, calculated as positive values) and exported to an Excel^®^ (Microsoft) sheet. Digital video recording of the trials were used to locate behaviors that might explain any artefacts (e.g., postures blocking marker tracking).

### 2.3. Procedures

Children with DCD were assessed on the MABC-2 (if not undertaken in the previous 6 months) and parents of both groups completed the DCDQ to confirm diagnosis. Parents also completed the CPRS. For the handwriting task, participants were seated on a height-adjustable chair with both feet touching the floor (or footplate) in front of a height-adjustable table and markers fitted. Position and height was set to ensure a stable seating position. A reference frame was obtained of baseline starting position with elbows flexed at a 90° angle with hands resting on the table surface. Paper was set at 10 cm from and parallel to the table edge with middle of the paper placed in body midline. If the child chose to rotate it according to his or her writing strategy, then this was allowed prior to measurements so as not to compel the subject to write differently than he or she was accustomed. Children completed the copying followed by the dictation task of the HHE.

### 2.4. Statistical Analyses

Statistical analyses were performed using SPSS (version 24, IBM Brøndby, Danmark ApS). Levene’s test for equality of variance was performed and non-parametric analyses were utilized if distributions of the dependent variables violated assumptions, recognizing that the t-test is robust to minor violations [32]. Between group comparisons using *t*-tests and Chi^2^ were made between age, gender, and attention. Means (standard deviations) and range (minimum and maximum) values were calculated for joint angles and angular acceleration for each child and for the groups. Medians and ranges were determined for criterion scores of handwriting legibility. Contrasts of handwriting tasks by group were undertaken using 2 × 2 (group by task, copying and dictation). Analysis of covariance (ANCOVA), adjusted for the differences in attention between groups, explored between-group differences in the movement parameters. Non-parametric analyses (Mann Whitney) were used to contrast handwriting legibility variables. Effect sizes (ES) were calculated as partial eta squared (p-η^2^) [33] and interpreted according to Miles and Shevlin [34], where p-η^2^ = 0.02 is considered a small ES, p-η^2^ = 0.13 represents a moderate ES, and p-η^2^ = 0.26 reflecting a large ES. Pearson (controlling for attention) and Spearman *rho* coefficient correlations explored the relationship between kinematic variables and handwriting performance. An alpha level of <0.05 was considered significant.

## 3. Results

**Figure 2:**
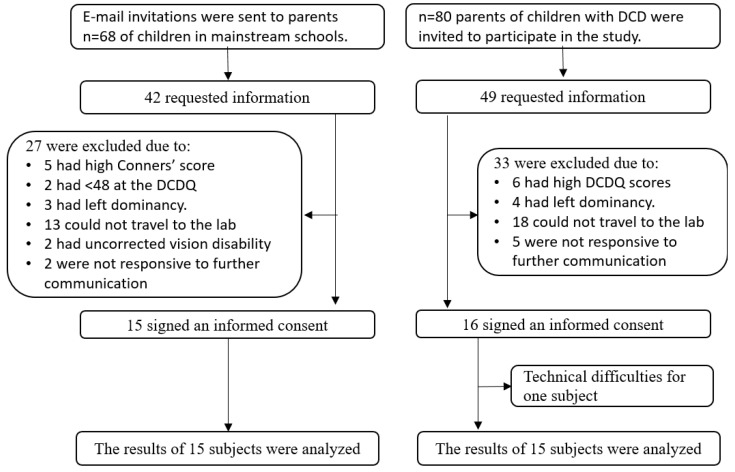
Allocation sequence and participant flow.

Participant recruitment is illustrated in Figure 2. The study population consisted of 14 boys and 16 girls, mean age 8 years one month (SD 11 months) attending classes in regular education (Table 1). Sixteen children diagnosed with DCD by the developmental doctor and with MABC-2 scores below the fifth percentile (5%) and DCDQ questionnaire scores ≤48 consented to participate. The motion analysis data from one male from this group failed to record and his data were excluded, yielding a total of 15 children with DCD. The control group then consisted of 15 TD volunteers. There were no differences between gender distribution (χ^2^ = 0.536 *p* > 0.5) nor parental education (fathers: χ^2^(1) = 0.16 *p* > 0.05; mothers: χ^2^(1) = 0.14 *p* > 0.05). Between-group differences were identified in the functional movement difficulties as reported in the DCDQ and number of hours spent writing per day, with children with DCD spending more time on writing tasks (Table 1). Differences in attention as recorded on the CPCS with children with DCD showed poorer attention and between-group comparisons were adjusted for attention where appropriate. Of note, no child with DCD met criteria for a co-morbid ADHD diagnosis.

### 3.1. Handwriting Performance

Significant differences were seen across all writing parameters in both copying and dictation. Legibility was significantly lower in the DCD group compared with the TD group in both copying (*U* = 12, *p* < 0.001)) and dictation (*U* = 20.6, *p* < 0.001) (See Table 2 for breakdown across legibility scores). Children with DCD were significantly less productive with fewer letters written per minute and increased overall time of writing (*p* < 0.003 across tasks; see Table 3). In view of the higher number of girls with DCD in our study, group by gender contrasts were run on handwriting performance and legibility in copying and dictation without significant effects (*p* > 0.05) and no further gender contrasts were run. We noted that one child in the TD group had relatively poor handwriting, although his scores for legibility and productivity were better than the scores of all but one child in the DCD group. Analyses were re-run excluding this child, without change to between-group contrasts on handwriting or movement kinematic parameters. We also re-ran the analyses excluding the one child with DCD whose writing at the limits of the TD children in legibility, albeit ≥2SDs slower, without change in results. We have therefore selected to report on the entire group as a more naturalistic representation of TD and DCD children.

Productivity (number of letters achieved by the second minute) showed a main effect of group (F (1,28) 29.1, *p* < 0.001, p-η^2^ = 0.509) and effect of task (F (1,28), 11.53, *p* = 0.002, p-η^2^ = 0.292) with no interaction effect; children with DCD were slower than the TD group and both groups were slower when writing from dictation (Table 2 and Table 3). Attention was not found to be a significant co-variate on any comparisons. Further comparisons contrasting overall rate of handwriting, e.g., contrasts between letters written in the first minute to those in the second, showed a difference between groups with children with DCD writing slower in the second minute in the dictation task while TD children wrote more quickly in the second minute (F (1,28) 4.65, *p* = 0.040). This trend was also seen in copying but did not reach significance (F (1,28) 3.20, *p* = 0.084) (Table 2). Contrasting copying to dictation, children produced more letters in dictation (t (29) −3.46, *p* = 0.002). Children with DCD made more deletions in both tasks (F (1,28) 22.96, *p* <.001). There was no group by task interactions, although TD children made marginally more deletions during dictation (mean difference 0.40) as opposed to the DCD group who made fewer deletions (mean difference −1.27).

### 3.2. Kinematic Parameters

**Figure 3:**
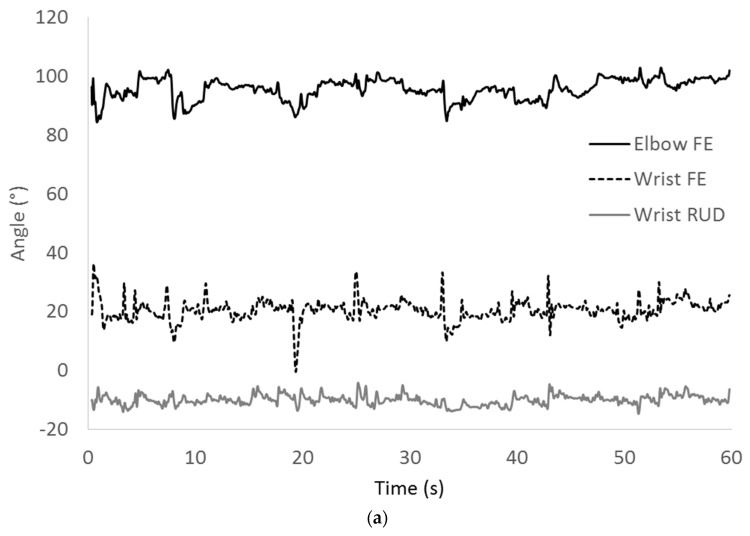

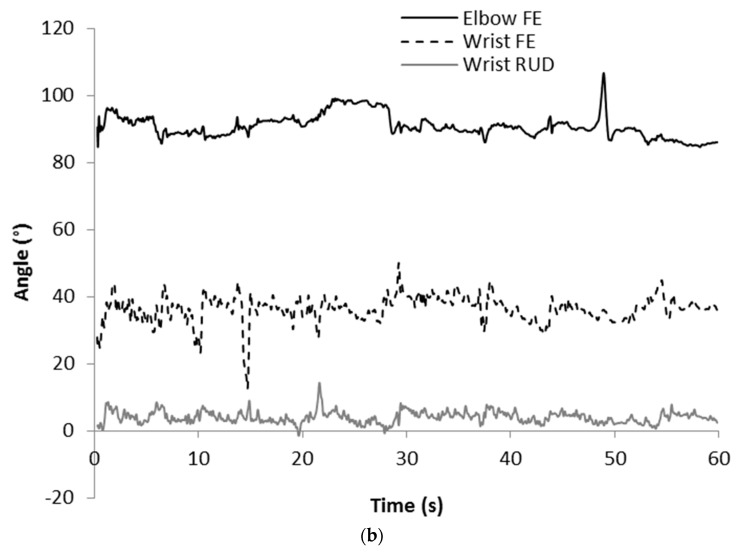
Exemplars of motion graphs during copying of (**a**) a child with developmental coordination disorder and (**b**) typically developing child. FE = flexion–extension; RUD = radial–ulnar deviation.

Kinematic parameters related to different aspects of handwriting performance. The differences between groups on kinematic parameters were only evident for radial ulnar deviation (wrist position) in dictation with children with DCD (F (1,27) 4.71, *p =* 0.04, p-η^2^ = 0.15) (Table 4). ANCOVA adjusting for attention did not show attention to be a significant covariate. Figure 3 illustrates the movements of the elbow and wrist (flexion–extension and radial–ulnar deviation) over a one-minute sample of writing during copying of a child from each group. Of note were apparent differences on visual analysis between the frequency of fluctuations of the elbow; a parameter that was not possible to measure without the synchronisation of the video camera to motion analysis to control for erasing. Different associations were seen between movement kinematics and handwriting performance between groups. Table 5 and Table 6 show the differing profiles of correlations between DCD and TD groups.

Greater wrist flexion and extension correlated significantly with greater legibility (lower values on the HHE global legibility scores) in both copying and dictation (*rho* −0.522 and −0.567, respectively) and also maintenance of right margin during copying (*rho* = 0.523) for children with DCD (Table 5). No significant correlations of wrist flexion and legibility were evident for TD children. Higher wrist mean flexion–extension acceleration, reflecting greater movement velocities, was significantly correlated with greater legibility in copying for the TD group (*rho* = −0.578). Greater elbow and smaller wrist acceleration were significantly associated with erasing in TD children (*rho* = 0.598 and *rho* = −0.544, respectively). In contrast, for children with DCD, only elbow acceleration approached a significant correlation with erasing text (*rho* = 0.505, *p* = 0.055), with wrist acceleration significantly correlated with maintenance of the right margin (note Hebrew text is written from the right towards the left margin).

Positive correlations were seen between joint ranges at the elbow and variations of acceleration for both groups; however, ranges and variations of movement speed between the elbow and wrist movements were not correlated for children with DCD. Whereas more inter-relationships between the speed changes and joint positions were evident across both proximal and distal joints for the TD group (Table 6). A differential impact of movement kinematics was also evident between groups.

## 4. Discussion

This study set out to identify differences in handwriting performance and movement characteristics of children with DCD compared to their TD peers across two different writing tasks; copying and dictation. As anticipated, children with DCD demonstrated poorer handwriting performance and efficiency as measured by the HHE; writing less legibly and more slowly during both copying and dictation tasks with large ESs across items. While we identified one child with poorer writing in the TD group (reflected in his legibility scores of 3 and 4 in copying and dictation, respectively), there was a notable absence of children with DCD who were close to typical for their age in handwriting in either copying or dictation (one only with relatively less poor legibility but still slow).

Comparisons between copying and dictation performance and productivity showed some interesting differences within and between groups. Consistent with the construct of the HHE, both groups were less legible (higher HHE scores) with reduced productivity (slower) in the copying task. It may have been anticipated that children with DCD would show greater difficulty in the dictation task due to reduced embedded representation (mental image) of letters for reproduction and thus greater reliance on the external reference for copying [7,35,36]. However unexplored differences in cross-modal integration (visual- versus auditory-motor integration) may have compensated for any deficits in embodied cognition in the DCD group in this age. Joint positions were similar between tasks although speed regulation differed for the most part; excluding elbow accelerations for the TD children which can be accounted for by the similarity in the number of erasing movements in the two tasks. Of note, however, was the slower performance of the children with DCD in the second minute compared with the first, particularly in the dictation task. This has implications for the accumulative decrement in handwriting performance of children with DCD, which may well be linked to the increased pauses, time not writing, that have been reported in this group during compositional writing [10,23], although it may also be associated with excessive time and energy expended on erasing errors. Increased pauses and erasing of errors may also be associated with characteristics of decision making, processes which have been associated with movement variability and variations in time to complete tasks [37]. With evidence of persistent complex executive function disorders in DCD [38], more detailed analyses of cognitive processes alongside kinematics of handwriting are warranted. Altered movement kinematics during prolonged handwriting have been associated with fatigue and corresponding compensatory processes in children with dysgraphia [39]. This may have particular implications in everyday life at school to ensure homework assignments at the end of the school day are dictated in short segments, allowing sufficient time for recording or provided by the teacher on a printed paper, or on the class webpage.

Contrary to expectation, there were no differences in motion parameters between the groups and only one significant difference in joint positions, with greater radial–ulnar deviation evident in the DCD group. There were, however, some interesting differences evident in the profiles of significant correlations between kinematic variables and handwriting legibility. Notably, the more extreme wrist flexion postures of children with DCD limited movement variation (acceleration and variance of movement speed), were associated with improved legibility. In contrast, a larger range of radial and ulnar wrist postures were associated with better writing in the TD group. Furthermore, the atypical profiles of wrist postures and variance in acceleration control were also associated with poor maintenance of margins in the DCD group. For both groups of children, joint ranges and positions of the two tasks were correlated.

Positive correlations were seen between joint ranges and acceleration for both groups, but for TD children this resulted in greater legibility of handwriting, whereas for children with DCD, the outcome was more likely to be a greater proportion of illegible text. Children with DCD were seen to have less control of movement variability for handwriting production as evidenced by the correlations between wrist acceleration and reduced numbers of legible letters, particularly in dictation with more restricted ranges of movement. This is in contrast to TD children, in whom greater variability of movement range and speed was associated with better handwriting. Furthermore, increased elbow movements with little wrist movements were associated with numbers of errors and subsequent erasing when copying, suggesting larger gross motor actions during erasing. In contrast, increased freedom of movement at the wrist with less elbow movements of TD children was associated with greater legibility representative of greater fluency. In children with DCD, wrist acceleration was negatively associated with forearm joint ranges, with larger maximal joint ranges associated with reduced movement, thereby potentially representing a more rigid pattern of movement and/or a fixation of joints at maximal ranges in some children at least.

Our results suggest that children with DCD use more extreme, end-of-range positions, which results in reduced legibility. This strategy may be attributed to increased stability by the reduction of the degrees of freedom, as evidenced through limited movement variations (reduced accelerations) across joints. This seems to be the antithesis of the movement profiles of TD children in whom increased movement variability was associated with better movement control and legibility of handwriting. Thus, rather than variability in movement supporting consistency in performance (legibility), children with DCD are needing to restrict or ‘freeze’ the degrees of freedom through maintaining more fixed-joint positions, particularly at the wrist, in order to produce more legible writing.

Control of movement variability is critical for developing skilled movements, with ‘noise’ being a general characteristic of the motor system. However, too much noise has been associated with poorer writing [40]. It is therefore notable that movement variability in postures and speed were associated with better legibility and productivity in the TD group, but for children with DCD, they were associated with illegibility and reduced productivity. Of note, in this respect, were our results reflecting less control over temporal parameters of movement in children with DCD, which were associated with poor legibility as well as attention to the handwriting rules (maintenance of margins). This is consistent with findings from Bo and colleagues who found children with DCD to have more difficulties in the temporal aspects of handwriting [21]. It is unclear however the extent to which this may be due to a lack of automaticity of movements for children with DCD and thus representative of problems in ‘dual task executive control’ [13,35,41,42] or issues of maturation over the ‘degrees of freedom’ problem. Children with DCD have been shown to have excessive variability with reduced accuracy across motor tasks, in comparison to TD children [43,44]. More specific associations between speed and trajectory in letter formation (printed and cursive letters ‘e’ and ‘l’) in a small group of children with movement difficulties (probable DCD) across a broader age range, were also seen to be linked to a greater variability in speed compared with their TD peers [22]. Neuroimaging of children with DCD has shown neural networks involved in predictive motor timing to be implicated, providing further support for deficits in temporal aspects of motor control in these children which may impact on handwriting [35,45]. A more recent meta-analysis of imaging findings in DCD provides additional support for the different patterns of brain activation in children with DCD [35], albeit with inconclusive findings regarding the automaticity deficit hypothesis as suggested by neural imaging of children with DCD with and without dyslexia [46]. Our results are consistent with this literature, with the pattern of movement acceleration across joints reflective of limited or more unpredictable fluctuations in movement speed impacting legibility with an overall reduction in speed and productivity.

Developmental theories regarding the control of the degrees of freedom suggest that early in motor development, too much variability is associated with greater performance variability [15]. This is followed by a period in which movements across different joints are restricted, ‘freezing of the degrees of freedom’ [15] to stabilize some performance parameters. More mature patterns of movement emerge later when movement variability is observed to facilitate consistent performance [47]. Although most of the kinematic measures did not differ between the two groups, the associations between postures and movement and handwriting performance were distinct. Additionally, while differences in attention control were evident between groups adjusting for attention did not have any significant impact on any parameter. Children with DCD appeared to constrain movement variability in order to achieve some consistency, albeit of poorer quality in handwriting performance and efficiency. In contrast, TD children seemed to be better able to harness movement variability for performance quality and productivity. Future studies should explore the zero-crossing of the angular velocity (fluctuations) at the elbow and wrist as an additional parameter associated with writing legibility and speed in children with DCD. The extent to which the degrees-of-freedom question relates to automaticity of motor control has yet to be explored in children with DCD.

It is unclear whether the lack of significant correlations between wrist and elbow movements when erasing during dictation may have been due to fewer errors erased in the TD children. Interestingly, the pattern of arm and wrist movements associated with erasing during copying suggested that children with DCD may use wrist movements, whereas TD children may recruit greater elbow movements. Although the motion-capture system was not able to detect specific episodes of erasing, video footage of the children reflects these observations. The implications of these different movement patterns are unclear without a more direct measure of the success of the erasing, to include also force and effort. Additionally, as noted above, report of potential attention problems differed between groups, but without impact on between-group comparisons. This may have reflected the fact that no child with DCD met criteria for an additional ADHD diagnosis. The combined effect of DCD and ADHD on handwriting may warrant further investigation in cases with more significant attention deficits.

Limitations of this study are reflected in the 4-point HHE scale measurement and small sample size, restricting in-depth analyses. Furthermore, although we undertook a number of comparisons, we did not correct for repeat analyses. This may have reduced the risk of a Type I error, although the level of significance of our findings in relation to the potential influence of movement kinematics on handwriting productivity suggests that additional Bonferroni adjustment would not necessarily change the interpretation of our findings with respect to handwriting performance [48]. The different outcomes of children with DCD compared with TD may also be influenced by other factors (e.g., different grip strategies and application of force). As such, our findings warrant corroboration through further studies. A further challenge was noted with the positioning of the markers to ensure clear sighting by the cameras. We had originally hoped to monitor posture in relation to upper limb and hand kinematics, but many children leant forward and blocked the markers. Furthermore, the limited distance between radial and ulnar styloids made tracking of forearm rotation a challenge. It was not possible to place small markers on the fingers to look at grip position as these were knocked off during writing. The use of active, rather than passive, markers may have assisted with postural analyses, but different marker designs are needed to address kinematics of finger movement control when interacting with objects.

We also note a gender bias with a more equal representation of girls and boys with DCD in our study. This may reflect recruitment rather than a sampling bias, with uptake to a fine motor handwriting project more likely to be preferred by girls than boys. However, we did not find an interaction effect of group and gender.

The contrast of movement during erasing between groups was limited by the small number of errors (erasing) in the TD group (mean 2.1 versus 7.3 in the DCD group). Nevertheless, the differing associations of movement kinematics with handwriting productivity and legibility provide insights into the mechanisms of motor control in children with DCD. Furthermore, there may have been subtle, smaller finger movements and differential influences of grip pressure and pencil pressure that were not detectable by the motion capture system. Future studies with detailed analyses to parse out effects of erasing would also provide interesting insights to the relative contribution of errors to overall productivity, legibility, and fatigue. Comparisons of movement kinematics (including the degrees of freedom question) and motor control, across fine and gross motor tasks, warrant more extensive research as a separate topic.

## 5. Conclusions

The differences in handwriting performance between children with and without DCD are evident in graphical quality and quantity, as well as in the kinematic parameters associated with legibility and productivity. The extent to which children with DCD constrain the degrees of freedom in handwriting and other skilled movement tasks warrants further investigation. Different sensor designs may be needed to examine kinematics of fine motor control. A greater understanding of movement characteristics of these children during handwriting may assist intervention design, including recommendations for keyboarding.

## Figures and Tables

**Table 1:** Participant characteristics (*n* = 30). Data of writing per hour, CPRS, and DCDQ scores are presented as means and standard deviation in parentheses.

	Children without DCD	Children with DCD	*T*-Test/ Chi^2 a^
Class group N (%)			
Year 2; 7 years	3 (20)	4 (27)	0.23 ^a^
Year 3; 8 years	6 (40)	6 (40)	
Year 4; 9 years	6 (40)	5 (33)	
Gender	8 male 7 female	6 male 9 female	0.54 ^a^
Writing hours per day	2.93 (0.46)	4.47 (0.92)	5.80 **
CPRS	14.80 (4.36)	22.60 (5.42)	4.34 **
DCDQ	72.07 (1.34)	15.40 (0.74)	144.0 **

^a^ = Chi^2^ ** *p* < 0.01 N = number; DCD = developmental coordination disorder; CPRS = Conners’ parent rating scale; DCDQ = developmental coordination disorder questionnaire.

**Table 2:** Handwriting legibility and letter formation. Data are presented as median and range in parentheses. Mann–Whitney U analyses were performed (Standardised U reported).

	Copying			Dictation		
	TD	DCD	U	TD	DCD	U
Legibility	1 (1–3)	3 (1–4)	−4.46 **	2 (1–4)	3 (2–4)	−3.97 **
Letter formation	1 (1–2)	3 (2–4)	−4.48 **	1 (1–2)	3 (1–4)	−4.39 **
Space between letters	1 (1–1)	3 (1–3)	−4.80 **	1 (1–1)	3 (1–3)	−4.80 **
Space between words	1 (1–4)	3 (1–4)	−3.70 **	2 (1–4)	3 (1–4)	−3.87 **
Font size	1 (1–2)	3 (1–3)	−4.77 **	1 (1–2)	2 (1–3)	−3.86 **
Right margin	1 (1–1)	2 (1–2)	−4.10 **	1 (1–1)	2 (1–3)	−4.06 **
Left margin	1 (1–1)	3 (2–4)	−5.06 **	1 (1–2)	2 (2–4)	−4.21 **
Total HHE score	8 (7–13)	20 (10–22)	−4.68 **	9 (7–15)	20 (9–22)	−4.30 **
Number of mistakes	2 (1–4)	6 (2–19)	−4.27 **	2 (1–4)	7 (2–12)	−3.77 **

** *p* < 0.01; HHE = Hebrew handwriting evaluation; DCD = developmental coordination disorder; TDTD = typically developing children.

**Table 3:** Handwriting productivity and speed. Data are presented as mean and standard deviation in parentheses.

		ANCOVA	DCD	TD
Copying	Number of letters in the 1st minute	16.5 (4.0)	10.7 (3.5)	F (1,28) 17.40, *p* < 0.001, partial η^2^ = 0.383
	Number of letters in the 2nd minute	18.1 (4.0)	10.1 (3.6)	F (1,28) 31.13, *p* < 0.001, partial η^2^ = 0.527
	Difference in productivity between 1st and 2nd minute	1.60 (3.72)	0.667 (3.2)	F (1,28) 3.20, *p* = 0.084
	Total time (s)	183.7 (47.2)	363.6 (183.5)	F (1,28) 13.51, *p* = 0.001, partial η^2^ = 0.326
Dictation	Number of letters in the 1st minute	18.9 (4.3)	13.2 (5.2)	F (1,28) 10.57, *p* = 0.003, partial η^2^ = 0.274
	Number of letters in the 2nd minute	19.9 (4.0)	11.9 (4.9)	F (1,28) 18.71, *p* < 0.001, partial η^2^ = 0.401
	Difference in productivity between 1st and 2nd minute	1.07 (2.99)	1.33 (3.11)	F (1,28) 2.16, *p* = 0.040
	Total time (s)	179.4 (52.9)	309.3 (91.3)	F (1,28) 22.74, I < 0.001 partial η^2^ = 0.448

DCD = developmental coordination disorder; TD = typically developing children.

**Table 4:** Kinematics of the two handwriting tasks: copying and dictation. Mean and standard deviation in parentheses (RUD of the wrist were very small, making the 2nd derivative, i.e., accelerations, too noisy, and therefore unreliable, and are thus not reported).

	Copying			Dictation		
	TD	DCD	F	TD	DCD	F
Max elbow extension [°]	106.94 (21.24)	109.13 (16.45)	0.089	106.81 (11.27)	111.41 (10.793)	1.08
Mean elbow FE range [°]	92.47 (21.07)	94.10 (10.72)	0.071	91.56 (10.575)	96.93 (13.284)	1.444
Max wrist flexion [°]	33.88 (12.78)	34.89 (12.55)	0.503	32.55 (13.87)	37.46 (14.96)	0.267
Mean wrist FE range [°]	21.23 (21.73)	16.44 (7.397)	0.670	15.02 (8.73)	20.98 (12.06)	2.30
Max RUD [°]	9.50 (8.61)	11.22 (4.45)	0.46	7.83 (6.17)	12.95 (6.26)	4.79 * p-η^2^ = 0.15
Mean RUD [°]	0.11 (5.77)	2.85 (5.83)	1.61	−0.24 (5.25)	2.98 (5.62)	2.54
Mean elbow Acc [°/s^2^]	0.24 (0.13)	0.24 (0.80)	0.008	−0.22 (0.15)	−0.59 (1.42)	0.965
Mean wrist FE Acc [°/s^2^]	0.01 (0.08)	0.06 (0.15)	2.06	0.07 (0.214)	0.08 (0.26)	0.013
SD elbow Acc [°/s^2^]	666.7 (153.9)	657.8 (110.6)	0.033	682.39 (91.9)	891.34 (866.07)	0.805
SD wrist FE Acc [°/s^2^]	183.0 (125.0)	198.9 (186.1)	0.072	193.70 (137.86)	275.28 (234.18)	1.28

* *p* < 0.05; DCD = developmental coordination disorder; TD = typically developing children; FE = flexion–extension, RUD = radial–ulnar deviation, Max = maximal, Min = minimal, Acc = acceleration, p-η^2^ = partial η^2^.

**Table 5:** Spearman correlations between handwriting legibility and movement kinematics (Spearman *rho* was used to compare interval and ordinal data. This did not allow for adjustment of differences in attention between groups. Attention was not found to be a significant co-variate in between group comparisons across variables and further adjustments were not considered necessary). Significant findings are in bold.

	Legibility Copy		Number Erased Copy		Legibility Dictated		Right Margin Copy	
	TD	DCD	TD	DCD	TD	DCD	TD ^a^	DCD
Max elbow ext [°]	−0.053	0.243	−0.143	0.214		−0.180	-	0.140
Mean elbow FE [°]	−0.079	−0.054	−0.171	−0.045	0.069	−0.028	-	0.384
Max wrist FE [°]	0.086	−0.417	−0.355	−0.123	0.089	−0.291	-	−0.523 *
Mean wrist FE [°]	0.252	−0.522 *	−0.316	−0.349	0.382	−0.567 *	-	0.481
Max RUD [°]	−0.399	0.013	−0.653 *	−0.445	0.232	−0.306	-	0.035
Mean RUD [°]	0.399	−0.066	−0.487	−0.420	0.476	−0.176	-	0.035
Mean elbow Acc [°/s^2^]	0.321	0.351	0.598 *	0.505 *	−0.063	0.192	-	0.244
SD elbow Acc [°/s^2^]	−0.186	−0.028	−0.362	−0.067	−0.185	0.376	-	0.209
Mean wrist FE Acc [°/s^2^]	−0.578 *	−0.010	−0.544 *	−0.183	−0.030	−0.110	-	−0.735 **
SD wrist FE Acc [°/s^2^]	0.651 **	−0.003	0.178	0.364	−0.063	−0.125	-	0.174

^a^ = insufficient range for value to be computed; ** *p* < 0.01, * *p* < 0.05; DCD = Developmental Coordination Disorder; TD = typically developing children; FE = flexion–extension, RUD = radial–ulnar deviation, Max = maximal, Min = minimal, Acc = acceleration.

**Table 6:** Pearson (partial) correlations between handwriting postures and kinematics.

		SD Elbow Acc [°/s^2^]		SD Wrist FE Acc [°/s^2^]		SD Wrist RUD Acc [°/s^2^]	
		TD	DCD	TD	DCD	TD	DCD
Copying	Max elbow extension [°]	0.940 **	0.593 *	0.311	0.169	0.382	0.195
	Elbow FE Range [°]	0.396	0.185	0.327	0.090	0.398	−0.036
	Mean elbow FE [°]	0.957 **	0.738 **	0.229	0.294	0.301	0.293
	Max Wrist Extension[°]	0.850 **	0.516 *	0.155	0.311	0.193	0.274
	Wrist FE range [°]	0.455	−0.203	0.651 *	−0.336	0.699 **	−0.314
	Mean wrist FE [°]	0.475	0.404	0.496	−0.344	0.488	−0.342
	Max RUD [°]	0.194	0.003	0.513	−0.283	0.484	−0.252
	RUD range [°]	0.254	−0.026	0.933 *	−0.129	0.970 **	−0.117
	Mean RUD [°]	−0.591	0.049	0.345	−0.095	0.327	−0.070
Dictation	Max elbow extension [°]	0.650 *	0.506	0.729 **	0.160	0.748 **	0.145
	Elbow FE Range [°]	−0.282	−0.309	0.266	0.719 **	0.226	0.741 **
	Mean elbow FE [°]	0.886 **	0.518 *	0.577 *	−0.138	0.626*	−0.147
	Max Wrist Extension [°]	0.622 *	0.220	0.224	−0.129	0.292	−0.191
	Wrist FE range [°]	0.310	−0.099	0.570 *	0.369	0.553 *	0.339
	Mean wrist FE [°]	0.606 *	0.216	0.179	−0.202	0.247	−0.279
	Max RUD [°]	0.510	0.021	0.035	0.211	0.048	0.198
	RUD range [°]	0.574 *	−0.011	0.818 **	0.144	0.810 **	0.149
	Mean RUD [°]	0.208	0.163	−0.295	0.004	−0.294	<0.001

** *p* < 0.01, * *p* < 0.05; DCD = developmental coordination disorder; TD = typically developing children; FE = flexion–extension, RUD = radial–ulnar deviation, Max = maximal, Min = minimal, Acc = acceleration.

## Data Availability

Data available on request from corresponding author.

## References

[1] Arighi P. (2016). The Power of Writing in Children. Arch. Argent. Pediatr..

[2] Velay J.L., Longcamp M. (2020). Motor Skills and Written Language Perception: Contribution of Writing Knowledge to Visual Recognition of Graphic Shapes. Language and Action in Cognitive Neuroscience.

[3] American Psychiatric Association (2013). Diagnostic and Statistical Manual of Mental Disorders.

[4] Asher A.V. (2006). Handwriting Instruction in Elementary Schools. Am. J. Occup. Ther..

[5] Dunford C., Street E., O’Connell H., Kelly J., Sibert J.R. (2004). Are Referrals to Occupational Therapy for Developmental Coordination Disorder Appropriate?. Arch. Dis. Child..

[6] Volman M.J.M., Van Schendel B.M., Jongmans M.J. (2006). Handwriting Difficulties in Primary School Children: A Search for Underlying Mechanisms. Am. J. Occup. Ther..

[7] Adams I.L.J., Lust J.M., Wilson P.H., Steenbergen B. (2017). Development of Motor Imagery and Anticipatory Action Planning in Children with Developmental Coordination Disorder—A Longitudinal Approach. Hum. Mov. Sci..

[8] Fuelscher I., Williams J., Wilmut K., Enticott P.G., Hyde C. (2016). Modeling the Maturation of Grip Selection Planning and Action Representation: Insights from Typical and Atypical Motor Development. Front. Psychol..

[9] Steenbergen B., Krajenbrink H., Lust J., Wilson P. (2020). Motor Imagery and Action Observation for Predictive Control in Developmental Coordination Disorder. Dev. Med. Child Neurol..

[10] Prunty M.M., Barnett A.L., Wilmut K., Plumb M.S. (2014). An Examination of Writing Pauses in the Handwriting of Children with Developmental Coordination Disorder. Res. Dev. Disabil..

[11] Prunty M.M., Pratt A., Raman E., Simmons L., Steele-Bobat F. (2020). Grip Strength and Pen Pressure Are Not Key Contributors to Handwriting Difficulties in Children with Developmental Coordination Disorder. Br. J. Occup. Ther..

[12] Berninger V.W., Rutberg J.E., Abbott R.D., Garcia N., Anderson-Youngstrom M., Brooks A., Fulton C. (2006). Tier 1 and Tier 2 Early Intervention for Handwriting and Composing. J. Sch. Psychol..

[13] Chang S.H., Yu N.Y. (2010). Characterization of Motor Control in Handwriting Difficulties in Children with or without Developmental Coordination Disorder. Dev. Med. Child Neurol..

[14] Schott N., El-Rajab I., Klotzbier T. (2016). Cognitive-Motor Interference during Fine and Gross Motor Tasks in Children with Developmental Coordination Disorder (DCD). Res. Dev. Disabil..

[15] Bernstein N. (1967). Coordination and Regulation of Movement.

[16] Revzen S., Koditschek D.E., Full R.J. (2009). Towards Testable Neuromechanical Control Architectures for Running. Adv. Exp. Med. Biol..

[17] D’Avella A., Bizzi E. (2005). Shared and Specific Muscle Synergies in Natural Motor Behaviors. Proc. Natl. Acad. Sci. USA.

[18] Green D., Chambers M.E., Sugden D.A. (2008). Does Subtype of Developmental Coordination Disorder Count: Is There a Differential Effect on Outcome Following Intervention?. Hum. Mov. Sci..

[19] Cuijpers R.H., van Schie H.T., Koppen M., Erlhagen W., Bekkering H. (2006). Goals and Means in Action Observation: A Computational Approach. Neural Netw..

[20] Barnett A., Henderson S., Scheib B., Schulz J. (2007). The Detailed Assessment of Speed of Handwriting (DASH). Manual. https://uhra.herts.ac.uk/handle/2299/11814?show=full.

[21] Bo J., Barta J., Ferencak H., Comstock S., Riley V., Krueger J. (2014). Developmental Characteristics in Cursive and Printed Letter-Writing for School-Age Children. J. Mot. Learn. Dev..

[22] Bo J., Colbert A., Lee C.M., Schaffert J., Oswald K., Neill R. (2014). Examining the Relationship between Motor Assessments and Handwriting Consistency in Children with and without Probable Developmental Coordination Disorder. Res. Dev. Disabil..

[23] Prunty M.M., Barnett A.L., Wilmut K., Plumb M.S. (2016). The Impact of Handwriting Difficulties on Compositional Quality in Children with Developmental Coordination Disorder. Br. J. Occup. Ther..

[24] Green D., Meroz A., Margalit A.E., Ratzon N.Z. (2012). A Validation Study of the Keyboard Personal Computer Style Instrument (K-PeCS) for Use with Children. Appl. Ergon..

[25] Keith Conners C., Sitarenios G., Parker J.D.A., Epstein J.N. (1998). The Revised Conners’ Parent Rating Scale (CPRS-R): Factor Structure, Reliability, and Criterion Validity. J. Abnorm. Child Psychol..

[26] Conners C.K. (2001). Conners’ Rating Scales Revised.

[27] Wilson B.N., Crawford S.G., Green D., Roberts G., Aylott A., Kaplan B.J. (2009). Psychometric Properties of the Revised Developmental Coordination Disorder Questionnaire. Phys. Occup. Ther. Pediatr..

[28] Henderson S., Sugden D., Barnett A. (2007). Movement Assessment Battery for Children, Movement ABC-2.

[29] Croce R.V., Horvat M. (2001). Reliability and Concurrent Validity of the Movement Assessment Battery for Children. Percept. Mot. Skills.

[30] Erez N., Parush S. (1999). The Hebrew Handwriting Evaluation 2 nd ed..

[31] Kasten P., Rettig O., Loew M., Wolf S., Raiss P. (2009). Three-Dimensional Motion Analysis of Compensatory Movements in Patients with Radioulnar Synostosis Performing Activities of Daily Living. J. Orthop. Sci..

[32] Howell D.C. (1995). Fundamental Statistics for the Behavioural Sciences.

[33] Levine T.R., Hullett C.R. (2002). Eta Squared, Partial Eta Squared, and Misreporting of Effect Size in Communication Research. Hum. Commun. Res..

[34] Miles J., Shevlin M. (2001). Applying Regression & Correlation: A Guide for Students and Researchers.

[35] Subara-Zukic E., Cole M.H., McGuckian T.B., Steenbergen B., Green D., Smits-Engelsman B.C.M., Lust J.M., Abdollahipour R., Domellöf E., Deconinck F.J.A. (2022). Behavioral and Neuroimaging Research on Developmental Coordination Disorder (DCD): A Combined Systematic Review and Meta-Analysis of Recent Findings. Front. Psychol..

[36] Deconinck F.J.A., Spitaels L., Fias W., Lenoir M. (2009). Is Developmental Coordination Disorder a Motor Imagery Deficit?. J. Clin. Exp. Neuropsychol..

[37] Hamilton L.D., Mazzo M.R., Petrigna L., Ahmed A.A., Enoka R.M. (2019). Poor Estimates of Motor Variability Are Associated with Longer Grooved Pegboard Times for Middle-Aged and Older Adults. J. Neurophysiol..

[38] Wilson P., Ruddock S., Rahimi-Golkhandan S., Piek J., Sugden D., Green D., Steenbergen B. (2020). Cognitive and Motor Function in Developmental Coordination Disorder. Dev. Med. Child Neurol..

[39] Kushki A., Schwellnus H., Ilyas F., Chau T. (2011). Changes in Kinetics and Kinematics of Handwriting during a Prolonged Writing Task in Children with and without Dysgraphia. Res. Dev. Disabil..

[40] van Galen G.P., Portier S.J., Smits-Engelsman B.C.M., Schomaker L.R.B. (1993). Neuromotor Noise and Poor Handwriting in Children. Acta Psychol..

[41] Nicolson R.I., Fawcett A.J. (2007). Procedural Learning Difficulties: Reuniting the Developmental Disorders?. Trends Neurosci..

[42] Pratt M.L., Leonard H.C., Adeyinka H., Hill E.L. (2014). The Effect of Motor Load on Planning and Inhibition in Developmental Coordination Disorder. Res. Dev. Disabil..

[43] Gomez A., Sirigu A. (2015). Developmental Coordination Disorder: Core Sensori-Motor Deficits, Neurobiology and Etiology. Neuropsychologia.

[44] Smits-Engelsman B.C.M., Wilson P.H. (2013). Age-Related Changes in Motor Imagery from Early Childhood to Adulthood: Probing the Internal Representation of Speed-Accuracy Trade-Offs. Hum. Mov. Sci..

[45] Debrabant J., Gheysen F., Caeyenberghs K., Van Waelvelde H., Vingerhoets G. (2013). Neural Underpinnings of Impaired Predictive Motor Timing in Children with Developmental Coordination Disorder. Res. Dev. Disabil..

[46] Biotteau M., Péran P., Vayssière N., Tallet J., Albaret J.M., Chaix Y. (2017). Neural Changes Associated to Procedural Learning and Automatization Process in Developmental Coordination Disorder and/or Developmental Dyslexia. Eur. J. Paediatr. Neurol..

[47] Kelso J.S. (2009). Synergies: Atoms of Brain and Behavior. Progress in Motor Control.

[48] Rothman K.J. (1990). No Adjustments Are Needed for Multiple Comparisons. Epidemiology.

